# Effect of long non-coding RNA highly up-regulated in liver cancer (HULC) on the prognosis of cancer: a meta-analysis

**DOI:** 10.18632/oncotarget.18452

**Published:** 2017-06-13

**Authors:** Yong Li, Yi-Hong Liu, Xian Chen, Yan-Juan Zhu, Hai-Bo Zhang, Yan Li, Jian-Ping Bai, Li-Rong Liu, Yan-Chun Qu, Xin Qu

**Affiliations:** ^1^ Department of Oncology, Guangdong Provincial Hospital of Chinese Medicine, Guangzhou 510120, Guangdong, China

**Keywords:** long non-coding RNAs, HULC, cancer, overall survival

## Abstract

Some studies investigated the association between highly up-regulated in liver cancer (HULC) and the overall survival (OS) of cancer. However, the results were conflicted and inconclusive. Therefore, we performed this meta-analysis to determine the association between HULC and the OS of cancer. A comprehensive online search was conducted on Online electronic databases (PubMed, EMBASE, and Wanfang database) from the earliest date to Aug 30, 2016. The strength of the association was calculated with the HRs and respective 95% CIs. The expression of HULC was significantly associated with OS of cancers (HR = 2.12; 95% CI 1.61 – 2.79; *P*<0.00001). In the subgroup analysis by ethnicity, the expression of HULC was significantly associated with OS in Chinese patients (HR = 2.04; 95% CI 1.55 – 2.70; *P*<0.00001). In the subgroup analysis by cancer type, HULC was associated with OS in osteosarcoma patients (HR = 3.36; 95% CI 1.02 – 11.07; *P =* 0.05) and in gastric cancer patients (HR = 2.17; 95% CI 1.08 – 4.38; *P =* 0.03). We performed the sensitivity analysis to assess the stability of the meta-analysis. A significant association was found in studies with adjustment (HR = 2.01; 95% CI 1.35 – 2.99; *P=* 0.0006). In conclusion, this meta-analysis suggested that high expression of HULC was significantly associated with OS of cancer.

## INTRODUCTION

Cancer is a disease, which come from interactions between genetic and environmental factors [1]. Although the target therapy and precision medicine have developed in some cancers, the outcome of most cancers is still poor and disappointing. Thus, it is urgent to find an useful biomarker of cancer prognosis.

long non-coding RNAs (lncRNAs) have important roles in biological processes. The role of lncRNAs is involved in many disease, including cancers [2]. For example, the expression of HOTAIR was a predictor of cancers [3].

Up-regulation of highly up-regulated in liver cancer (HULC) has been detected in many human malignancies [4]. Some studies investigated the association between HULC and the survival of cancer [5–11]. However, the results were conflicted. Therefore, we did this meta-analysis to investigate the association between HULC and the survival of cancer.

## RESULTS

### Literature search

691 patients with cancer were included in this meta-analysis. All the studies were cohort studies. There were 6 studies of Asian populations and 1 study of American population. The cancer types included pancreatic cancer, osteosarcoma, hepatocellular carcinoma, gastric cancer, and diffuse large B-cell lymphoma. The sample sizes included from 33 to 304. All the studies provided overall survival (OS) data. The NOS were high, suggesting that the quality of the included studies were well. The characteristics of each study are presented in Table 1.

**Table 1:** Characteristics of the included studies

First author	Year	Study design	Study location	Race	Cancer type	Tumor stage	Samle size	Outcome	Cutoff value	Analytical method	Covariants	HR and 95%CI	NOS
Peng	2014	Cohort	China	Chinese	PC	NA	304	OS	NA	Multivariate	Tumor size, lymph node metastasis, vascular invasion	2.84 (1.33-6.06)	8
Sun	2015	Cohort	China	Chinese	Osteosarcoma	II-III	78	OS	Median	Multivariate	Age, gender, tumor size, location, clinical stage, metastasis	2.28 (1.48-3.51)	8
Li	2016	Cohort	China	Chinese	HCC	I-IV	38	OS	Fold change	NA	No	2.22 (0.55-8.96)	7
Jin	2016	Cohort	China	Chinese	GC	I-IV	54	OS	Fold change	NA	No	2.14 (0.77-5.95)	7
Zhang	2016	Cohort	China	Chinese	GC	NA	42	OS	Fold change	NA	No	2.20 (0.84-5.76)	7
Uzan	2016	Cohort	Brazil	American	Osteosarcoma	NA	33	OS	NA	Univariate	No	8.72 (1.50-50.70)	7
Peng	2016	Cohort	China	Chinese	DLBCL	I-IV	142	OS	NA	Multivariate	B symptoms, CHOP-like treatment, Rituximab, IPI, Ann arbor stages	2.84 (1.33-6.06)	8

PC, pancreatic cancer; HCC, hepatocellular carcinoma; GC, gastric cancer; DLBCL, diffuse large B-cell lymphoma; OS, overall survival; NOS, Newcastle-Ottawa Scale; NA, not available.

### Main result

**Figure 1:**
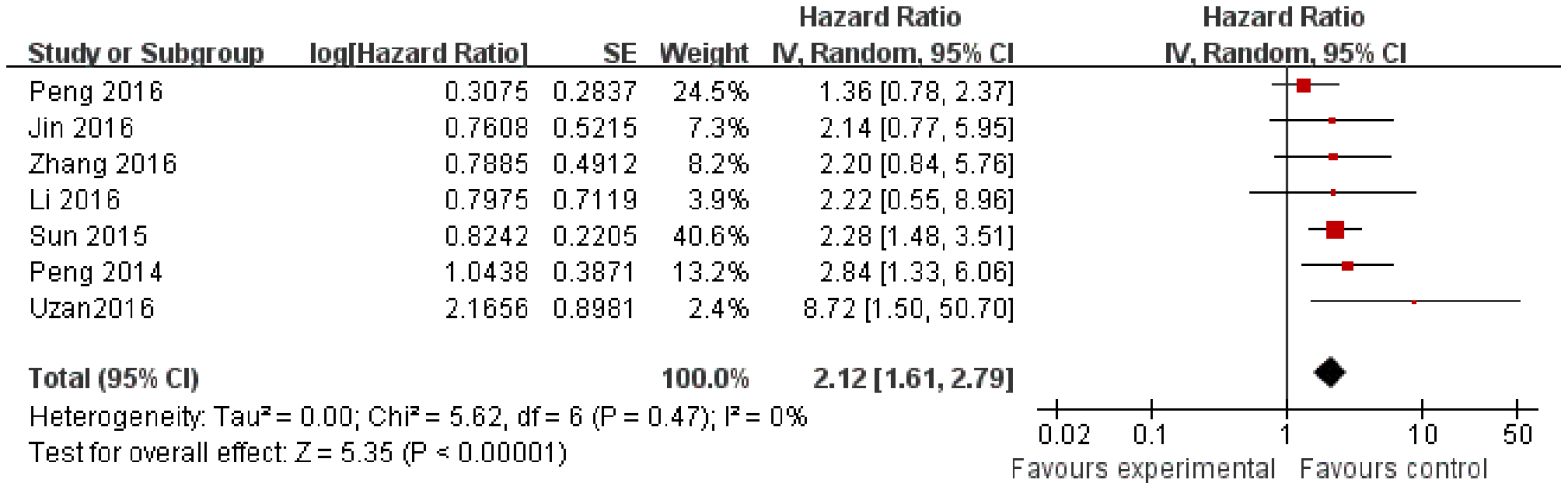
The association between HULC and OS in cancer

As shown in Figure 1, the expression of HULC was significantly associated with OS of cancers (HR = 2.12; 95% CI 1.61 – 2.79; *P*< 0.00001).

### Subgroup analysis by ethnicity

**Figure 2:**
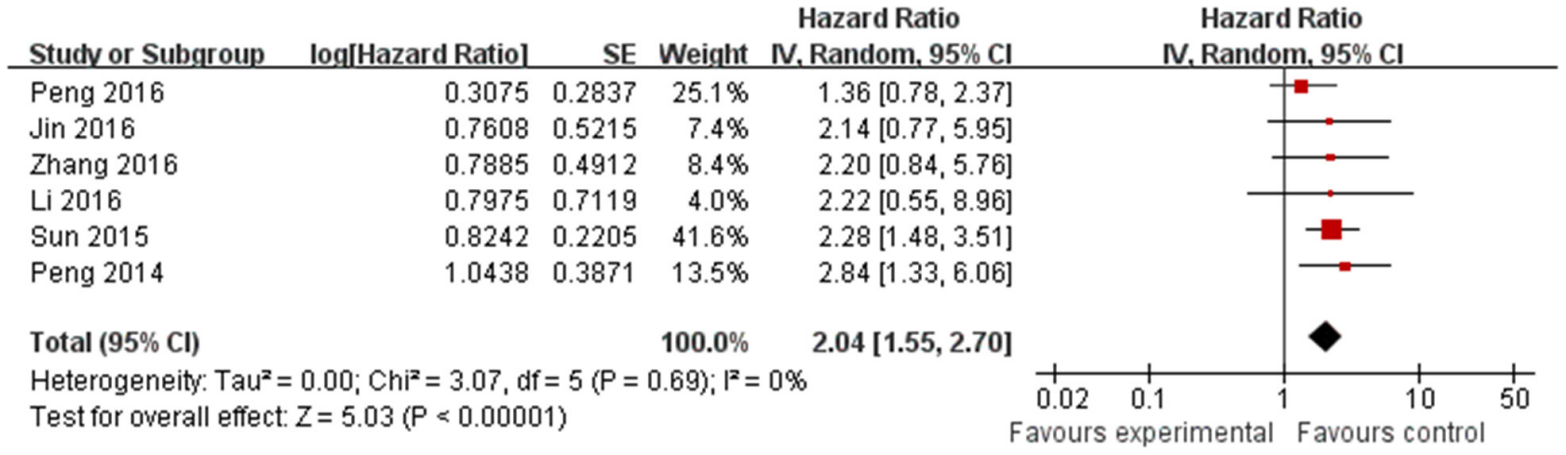
The association between HULC and OS in cancer in Chinese patients

In the subgroup analysis by ethnicity, the expression of HULC was significantly associated with OS in Chinese patients (HR = 2.04; 95% CI 1.55 – 2.70; *P*< 0.00001; Figure 2).

### Subgroup analysis by cancer type

**Figure 3:**
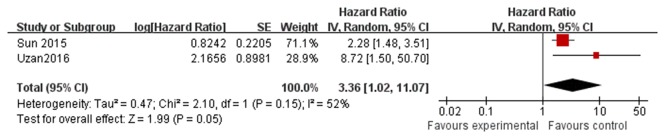
The association between HULC and OS in osteosarcoma

**Figure 4:**
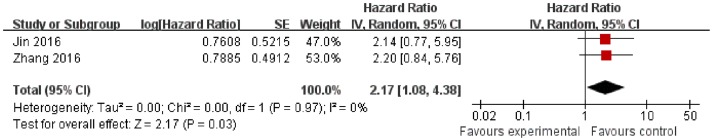
The association between HULC and OS in gastric cancer

In the subgroup analysis by cancer type, HULC was associated with OS in osteosarcoma patients (HR = 3.36; 95% CI 1.02 – 11.07; *P=* 0.05; Figure 3) and in gastric cancer patients (HR = 2.17; 95% CI 1.08 – 4.38; *P=* 0.03; Figure 4.

### Sensitivity analysis and publication bias analysis

**Figure 5:**
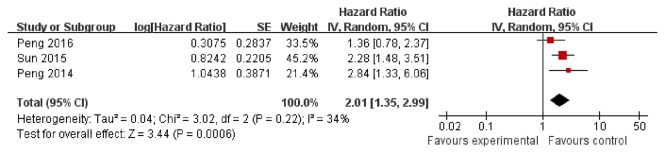
The adjusted result of the association between HULC and OS in cancer

The sensitivity analysis was assessed to see the stability of the meta-analysis. A significant association was found in studies with adjustment (HR = 2.01; 95% CI 1.35 – 2.99; *P=* 0.0006; Figure 5).

**Figure 6:**
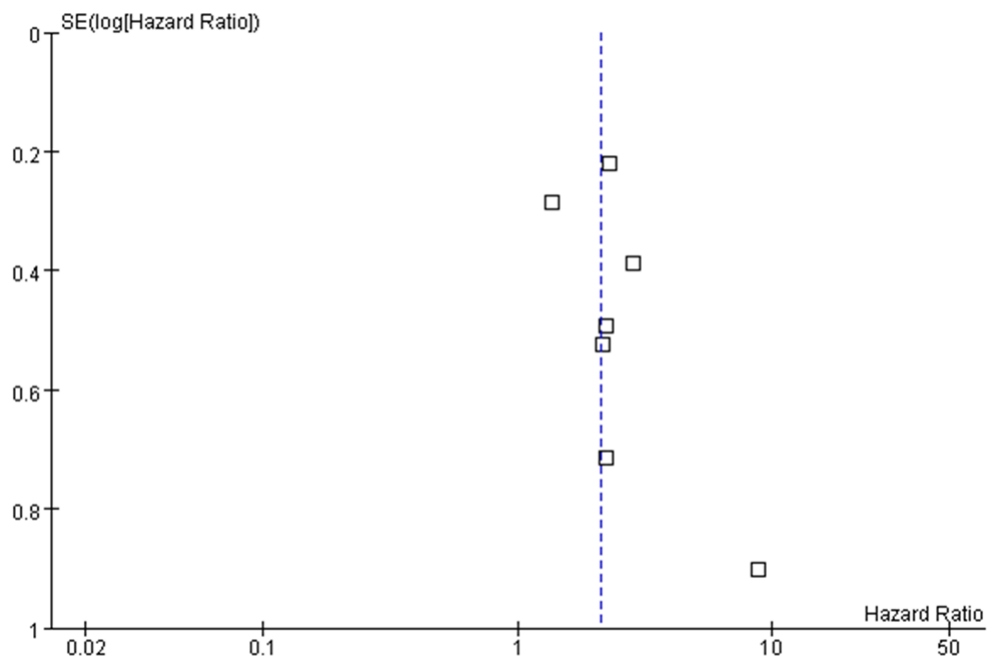
The funnel plot of the association between HULC and OS in cancer

The s funnel plot was symmetrical (Figure 6). In addition, no significant publication bias was detected by Begg’s test (*P* = 0.272).

## DISCUSSION

This meta-analysis with 691 cancer patients investigated the association between HULC and the survival of cancer. Results from this meta-analysis revealed that cancer patients with HULC might have shorter OS. Furthermore, Asians patients with HULC showed increased shorter OS in the subgroup analysis. Only one study using American patient included in this meta-analysis, thus more studies with other populations should be conducted to test this result. In the subgroup analysis by cancer type, we found a positive association in osteosarcoma patients and gastric cancer patients.

Wang et al. suggested HULC was an endogenous 'sponge'. The HULC down-regulates some microRNAs activities [12]. Kang et al. reported that HULC rs7763881 A/C polymorphisms could decrease the risk of esophageal cancer [13]. Cui and colleagues found that HULC was an oncogene in hepatoma cells. It could deregulate lipid metabolism [14]. Zhao et al. suggested that HULC was associated with lymph node and distant metastasis [4]. Xie et al. indicated that HULC could be a biomarker for the prognosis of hepatocellular carcinoma [15].

Some limitations should be addressed. First, another studies are needed to determine the results among different races. Second, publication bias cannot be ruled out. Third, we did not perform other subgroup analyses due to limited data.

In conclusion, this meta-analysis suggested that HULC was a biomarker of the prognosis of cancer.

## MATERIALS AND METHODS

### Publication search

A comprehensive online search was conducted on Online electronic databases (PubMed, EMBASE, and Wanfang database) from the earliest date to Aug 30, 2016. The words (or carcinoma) and (highly up-regulated in liver cancer or HULC) were searched, with no language or other restrictions. The whole text was reviewed if information in title or abstract is not sufficient to make a decision. Secondary searches of literature were conducted by searching the reference lists of the selected studies and relevant reviews to avoid missing.

### Inclusion and exclusion criteria

Studies were included if they met the following criteria: [1] case–control or cohort study; [2] about the association between HULC and the survival of cancer; and [3] had available data or could be calculated from the paper. Accordingly, the exclusion criteria were [1] duplicate data, [2] reviews, abstracts, case report, or animal studies.

### Data extraction

The following data were recorded from each article: first author, year, race, cancer type, numbers of patients, outcome, parameters used for adjustment, study design, study location, tumor stage, cutoff value, and analytical method. The data were extracted by two of the authors independently. Discrepancies between these two authors were resolved by discussion. The included studies were assessed independently by the two reviewers using the Newcastle-Ottawa Scale (NOS).

### Statistical analysis

The strength of association between HULC and the survival of cancer was estimated by HR with corresponding 95% CI. Q-statistic was applied to investigate heterogeneity among studies. P-value greater than 0.1 for Q test suggested a lack of statistically significant heterogeneity. The random-effect model (DerSimonian-Laird method) was used. In addition, the I^2^-test was employed to accurately measure the degree of heterogeneity. Furthermore, the I^2^-value less than 25% was equivalent to mild heterogeneity, and values between 25% and 50% was equivalent to moderate heterogeneity, whereas values greater than 50% was equivalent to large heterogeneity among studies. Potential publication bias was estimated by symmetry of funnel plot of HR versus the standard error of log (HR) and the visual symmetrical plot indicated that there was no publication bias among studies. Stratified analyses were conducted in terms of race and cancer type. All statistical tests in this meta-analysis were two-tailed and P-value ≤ 0.05 was considered statistically significant unless otherwise noted. All statistical analyses were performed with Revman 5.1 software (Nordic Cochrane Center, Copenhagen, Denmark) and STATA 11.0 software (Stata Corporation, College Station, TX).
